# Retinal Pre-Conditioning by CD59a Knockout Protects against Light-Induced Photoreceptor Degeneration

**DOI:** 10.1371/journal.pone.0166348

**Published:** 2016-11-28

**Authors:** Delu Song, Brooks Wilson, Liangliang Zhao, Rupak Bhuyan, Mausumi Bandyopadhyay, Arkady Lyubarsky, Chen Yu, Yafeng Li, Levi Kanu, Takashi Miwa, Wen-Chao Song, Silvia C. Finnemann, Bärbel Rohrer, Joshua L. Dunaief

**Affiliations:** 1 The F.M. Kirby Center for Molecular Ophthalmology, Scheie Eye Institute, Perelman School of Medicine at University of Pennsylvania, Philadelphia, PA; 2 Department of Ophthalmology, Medical University of South Carolina, Charleston, SC; 3 Department of Ophthalmology, The Second Hospital of Jilin University, Jilin, China; 4 Center for Cancer, Genetic Diseases, and Gene Regulation, Department of Biological Sciences, Fordham University, Bronx, NY; 5 Department of Pharmacology and Translational Therapeutics, Perelman School of Medicine, University of Pennsylvania, Philadelphia, PA; 6 Research Service, Ralph H. Johnson VA Medical Center, Charleston, SC; University of Florida, UNITED STATES

## Abstract

Complement dysregulation plays a key role in the pathogenesis of age-related macular degeneration (AMD), but the specific mechanisms are incompletely understood. Complement also potentiates retinal degeneration in the murine light damage model. To test the retinal function of CD59a, a complement inhibitor, CD59a knockout (KO) mice were used for light damage (LD) experiments. Retinal degeneration and function were compared in WT versus KO mice following light damage. Gene expression changes, endoplasmic reticulum (ER) stress, and glial cell activation were also compared. At baseline, the ERG responses and rhodopsin levels were lower in CD59aKO compared to wild-type (WT) mice. Following LD, the ERG responses were better preserved in CD59aKO compared to WT mice. Correspondingly, the number of photoreceptors was higher in CD59aKO retinas than WT controls after LD. Under normal light conditions, CD59aKO mice had higher levels than WT for GFAP immunostaining in Müller cells, mRNA and protein levels of two ER-stress markers, and neurotrophic factors. The reduction in photon capture, together with the neurotrophic factor upregulation, may explain the structural and functional protection against LD in the CD59aKO.

## Introduction

Age-related macular degeneration (AMD) is the leading cause of irreversible blindness among people over 55 years old [1]. It has two forms: the non-exudative (dry) form, which is more prevalent, and the exudative (wet) form, which is more acutely damaging. Although our understanding of contributing mechanisms remains incomplete, genetic and histological evidence indicate that elevated complement activation is involved [2]. In addition, oxidative stress has been implicated[3].

Light-induced photoreceptor degeneration has been studied in experimental animals for over 40 years as a model of oxidative stress-induced photoreceptor degeneration [4–5]. During light damage (LD), photo-oxidative stress combined with high oxygen tension and a high concentration of easily oxidizable polyunsaturated fatty acids injures photoreceptor cells. The complement system has been implicated in LD-induced photoreceptor degeneration. Eliminating the alternative pathway by knockout of complement factor D has been shown to protect photoreceptors from LD in Balb/c mice [6]. Further, complement genes are up-regulated in the retina following light damage [7], and complement-expressing microglia/macrophages infiltrate the photoreceptor layer following LD [8–9].

As a part of innate immunity, complement plays an important role in host defense. Activation of the complement system must be carefully controlled by complement regulatory proteins in the fluid phase and on cell membranes. CD59 is a membrane complement regulatory protein that is attached to the cell membrane via a glycosylphosphatidylinositol (GPI) anchor. CD59 inhibits the formation of C5b-9 (membrane attack complex; MAC) by preventing the binding of C9 to the nascent C5b-8 complex [10–13].

CD59 protein is localized to the basolateral surface of retinal pigment epithelium (RPE) cells [14]. CD59 expression is relatively low in young and healthy RPE, but later increases with age [15]. Interestingly, CD59 levels in AMD patients are decreased relative to age-matched controls in flattened RPE cells overlying drusen and near regions of geographic atrophy [16]. In contrast, choroidal levels remain unchanged. This reduction in surface RPE expression of CD59 is mimicked in human RPE cells in culture exposed to oxidative stress induced by H_2_O_2_ [14] or by phagocytosis of bisretinoid-laden photoreceptor outer segment fragments [17]. Furthermore, there is a negative correlation between the amount of MAC deposition and the number of RPE cells [18]. These results suggest an important role of CD59 in limiting the amount of complement activation in AMD. While knockout of CD59a in mice has not been shown to cause retinal degeneration, it has been associated with upregulation of alternative complement pathway activators in the retina, and especially the RPE [19]. Herein, we investigated whether constitutive lack of CD59a in CD59aKO mice would increase photoreceptor susceptibility to LD. Surprisingly, we found protection against LD in CD59a KOs and uncovered evidence of retinal pre-conditioning.

## Materials and Methods

### Animals and light exposure

Male CD59aKO Balb/c mice, aged 12 weeks, were generated and housed at the University of Pennsylvania [20]. Age- and sex-matched control wild-type (WT) Balb/c mice were purchased from Jackson Laboratory (Bar Harbor, ME, USA) and maintained in adjacent cages to CD59aKO mice in the UPenn animal facility for at least one week before light exposure. The Rpe65 variant was checked in genomic DNA from CD59aKO mice and found to be the same as WT Balb/c controls, with Leu at amino acid 450. All mice were maintained in a temperature-controlled room at 21–23 C with a 12 h:12 h light-dark photoperiod. For light damage (LD), mice were exposed to 10k lx of cool white fluorescent light continuously for 8 h in a room that was well-ventilated. During this period, mice had free access to water and a standard laboratory diet. After light exposure, mice were returned back to normal light/dark cycle for 7 days. Eyes were enucleated after sacrifice at day 7 following LD for morphologic analysis. Euthanasia was performed by ketamine/xylazine administration followed by cervical dislocation. The euthanasia methods are consistent with the recommendations of the American Veterinary Medical Association (AVMA) Guidelines on Euthanasia. A separate set of experiments was performed on C57BL/6 male and female CD59a deficient mice kindly provided by Dr. B. Paul Morgan (Cardiff University, UK) [21], which were confirmed to be positive for the Met variant of Rpe65 at amino acid 450. These mice were raised at the Medical University of South Carolina animal facility with a 12 h:12 h light-dark photoperiod. For LD, mice were dark-adapted overnight, pupils dilated and exposed to 30k lx of cool white fluorescent light in a well-ventilated room continuously for 4 h. After light exposure, mice were returned to a dark room for 10 days. Experimental procedures were performed in accordance with the Association for Research in Vision and Ophthalmology (ARVO) statement for the use of animals in ophthalmology and vision research. All protocols were approved by the animal care review board of the University of Pennsylvania and the Medical University of South Carolina.

### Immunofluorescence

Mice were humanely euthanized, and eyes enucleated and immersion-fixed in 4% paraformaldehyde (PFA) for 10 min. Eyeballs were then rinsed in PBS, and eye cups were generated by removing the anterior segment. The eye cups were infiltrated in 30% sucrose overnight and embedded in Tissue-Tek OCT (Sakura Finetek, Torrance, CA, USA). Immunostaining for CD59a was performed on 10-μm-thick cryostat sections. The primary antibodies were mouse anti-mouse CD59a (HM1116, Hycult, PA, USA) at 1:500 dilution and a mouse-on-mouse polymer IHC kit (ab127055, Abcam, MA, USA) was used to optimize signal to background staining ratio. We used an HRP-conjugated secondary antibody followed by diaminobenzidine to yield a brown precipitate. Rat anti-mouse glial fibrillary acid protein (GFAP) antibody (13–0300, Invitrogen, Camarillo, CA, USA) was used at a 1:500 dilution. GFAP antibody was detected using fluorophore-labeled secondary antibodies (Jackson ImmunoResearch Laboratories, Inc., PA, USA). Control sections were treated identically but without primary antibody. The sections were analyzed by fluorescence microscopy with identical exposure parameters (model TE300 microscope, Nikon, Tokyo, Japan) with ImagePro software (Media Cybernetics, Silver Spring, MD, USA).

### Morphologic analysis

Eyes enucleated 7 (Balb/c) or 10 (C57BL/6) days following LD were immersion-fixed in 2% paraformaldehyde/2% glutaraldehyde overnight. For standard histology, 3-μm-thick plastic sections were cut in the sagittal plane and toluidine blue-stained as we have described [6,22]. The number of nuclei per column of outer nuclear layer (ONL) photoreceptors was counted in triplicate at four locations, 400 μm away from the ONH and 400 μm from the ora serrata, using image analysis software (ImagePro Plus 4.1; Media Cybernetics) to calculate distances from manually set lengths.

### Quantitative real-time PCR

Gene expression in the neurosensory retina (NSR) samples obtained from CD59aKO and WT control mice was analyzed by quantitative RT-PCR as we have described [23]. Quantitative PCR (qPCR) with Taqman probes (ABI, Grand Island, NY, USA) was performed using a DNA amplification/detection system (Prism model 7500; ABI) with the ΔΔCT method, which provides normalized expression values. Probes used were cd59a (CD59a, Mm01276239_m1), rhodopsin (Rho, Mm01184405_m1), fibroblast growth factor 2 (Fgf2, Mm00433287_m1), vascular endothelial growth factor (Vegf, Mm01281449_m1), glucose regulated protein 78 (Grp78, Mm00517690_g1), and glucose regulated protein 94 (Grp94, Mm00441926_m1). To quantify the expression of target genes, eukaryotic 18S rRNA (Hs99999901_s1) was used as an endogenous control. The amount of target mRNA was compared among the groups of interest. All reactions were performed on samples from four mice. For each mouse, technical triplicates were analyzed.

### Electroretinography

Electroretinography (ERG) recordings for Balb/c mice followed procedures described previously [24]. In brief, mice were dark-adapted overnight and then anesthetized. Pupils were dilated with 1% tropicamide (Mydriacyl; Alcon), and mice were placed on a stage maintained at 37°C. Two electrodes made of UV-transparent plastic with embedded platinum wires were placed in electrical contact with the corneas. A platinum wire loop placed in the mouth served as the reference and ground electrode. The ERGs were then recorded (Espion Electrophysiology System; Diagnosys LLC, Lowell, MA, USA). The apparatus was modified by the manufacturer for experiments with mice by substituting light-emitting diodes with emission maximum at 365 nm for standard blue ones. The stage was positioned in such a way that the mouse's head was located inside the stimulator (ColorDome; Diagnosys LLC), thus ensuring uniform full-field illumination. The flash intensities of rod a- and b-waves are 0.01 and 500 scot cd m^-2^s, respectively.

ERGs recorded on C57BL/6 mice were performed according to our published procedures [25]. Mice were dark-adapted overnight, anesthetized using xylazine (20 mg/kg) and ketamine (100 mg/kg) and pupils were dilated with a drop of phenylephrine HCl (2.5%) and tropicamide (1%). Body temperature was stabilized via a DC-powered heating pad, and held at 37°C. A needle ground electrode was placed in the tail and a reference needle electrode in the forehead. ERG responses were measured using a contact lens containing a gold-ring electrode [26] held in place by a drop of methyl cellulose. ERGs were recorded with the UTAS-2000 (LKC Technologies, Inc., Gaithersburg, MD) system, using a Grass strobe-flash stimulus. The responses were recorded at a gain of 2 k using a notch filter at 60 Hz, and were bandpass filtered between 0.1 and 1500 Hz. ERGs were recorded in response to a single light flash (2.48 photopic cd-s/m2 at the dome's inner surface as calibrated by the manufacturer; in units of time-integrated luminance). Rod a-wave amplitudes were measured from baseline to a-wave trough; b-wave amplitudes were measured from a-wave trough or baseline to peak of b-wave.

### Western blotting analysis

Retinas were dissected and frozen immediately at −80°C, then lysed in RIPA buffer plus Complete, EDTA-free protease inhibitor (Roche Diagnostics, Indianapolis, IN). Equal amounts of total protein were used per lane. Native samples were used for detection with anti-Rhodopsin antibody and denatured samples (incubated for 10 m at 70°C) were used for detection with anti-KDEL antibody. Protein lysates were separated on a 4–12% gradient SDS-PAGE gel and transferred to a nitrocellulose membrane. Blocking was achieved by incubation for 1 hour in Tris-buffered saline that contained 5% milk and 0.1% Tween 20. Membranes were incubated overnight at 4°C with anti-Rhodopsin antibody (ab5417, Abcam, Cambridge, MA, USA) at 1:1000 dilution and 4 μg/mL mouse anti-KDEL antibodies (ab12223, Abcam, Cambridge, MA, USA). After washes, membranes were incubated with anti-mouse secondary antibody at a 1:5000 dilution (Jackson ImmunoResearch Laboratories, Inc.) and were developed with ECL Plus enhanced chemiluminescence reagent (GE Healthcare, Chalfont St. Giles, UK). Anti–actin antibody (Sigma-Aldrich, St. Louis, MO) was used as loading control. Images were acquired with the Amersham Imager 600 (GE Healthcare), and densitometry analysis was performed with Image J.

### 11-*cis* retinal regeneration analysis

After overnight dark-adaption, pupils were dilated with topical 1% phenylephrine and 1% atropine, and the mice were placed in a cubic plastic box lined with aluminum foil; this design allowed for uniform full-field illumination of the animals. The light source utilized in experiments used 10 halogen bulbs (12 V 50 W each; Bulbtronics, Farmingdale, NY), and produced Ganzfeld illuminance of 4×10^4^ scot cd m^-2^; the mice were illuminated at this light intensity for 6 s. Since the rhodopsin bleach rate in Balb/c mice in a Ganzfeld is of 8.3×10^−6^ s^-1^ (scot cd^-1^ m^2^)^-1^ [27], in our experiments (illuminance of 4×10^4^ scot cd m^-2^), the rhodopsin bleach rate was 8.3×10^−6^×4×10^4^ s^-1^ ~ 3 s^-1^, and the corresponding time constant was ~ 0.3 s, so a 6 s illumination guaranteed photoisomerization of >99.99% of rhodopsin.

After illumination, mice were euthanized at predetermined time points, and their eyes were flash frozen in dry ice. Retinoid extractions for analysis of retinoid content in the course of regeneration after illumination was performed following a modified lipid-extraction protocol [28] as we described and validated previously [29]. One or two frozen mouse eyes were placed in a centrifuge tube on dry ice, 180 μL dissection medium was added (vol/vol: 60% MeOH, 30% chloroform, 10% 1 M NH_2_OH; pH 7), the eyes were minced, and medium was added to 0.6 mL final volume. Samples were cooled on dry ice for 3 to 5 m, and sonicated with a microtip probe (30 pulses at 0.3 power and 0.1 duty cycle; Branson, Danbury, CT). Temperature in the sample was monitored with a BAT-10 digital thermometer equipped with an HT-1 thermocouple (Physitemp, Clifton, NJ); at the end of sonication, it did not exceed 150°C. Chloroform (200 μL) was added, samples were vortexed for 5 s, then 250 μL of water was added, and samples were vortexed and then centrifuged at 14,000 rpm for 5 m. The chloroform (lower) phase was collected, dried under nitrogen, and dissolved in hexane (200 μL). Normal phase HPLC analysis of retinoids was performed on a model 1100 HPLC system equipped with multidiode array detector and Chemstation software (Agilent, Palo Alto, CA) in the following system: Supelcosil LCSi analytical column (4.6 x 150 mm, 3-μm particle size, Sigma-Aldrich, St. Louis, MO) and 92.5:7.5 n-hexane/ethyl acetate mobile phase. The retinoids eluted were identified by their absorbance spectra. All chemicals were from Sigma-Aldrich (St. Louis, MO). All procedures were performed under dim red light.

### Rhodopsin measurements

Retinas from dark-adapted mice were collected under dim red light. The samples were prepared according to our previously published method [30]. In short, tissues were homogenized in 500 μl PBS containing 1X protease inhibitor (Sigma-Aldrich; St. Louis, MO). Samples were centrifuged (27,000× g, 15 min), the supernatant discarded and the remaining pellet solubilized in 1% dodecylmaltoside (in sodium phosphate buffer, pH 7.4). The sample was shaken at 4°C for 2 h, centrifuged (88,000× g for 10 min), and measured on a Softmax Pro spectrophotometer (Varian; Palo Alto, CA). The difference spectra were determined from measurements before and after bleaching with white light. The concentration of rhodopsin was calculated using the following extinction coefficient: ε (rhodopsin) = 40,600 l·mol^−1^·cm^−1^.

### RPE phagocytosis assay via immunostaining with anti-rhodopsin antibody

RPE phagocytosis was assessed using an established procedure described in detail previously [31]. Briefly, eyes were enucleated from mice sacrificed 1 or 2.5 h after lights turned on or 1 h after lights turned off and immediately immersion fixed in Davidson’s fixative (33% ethanol, 22% formalin, and 11.5% acetic acid). Following removal of the anterior segment, eye cups were embedded in paraffin. 7-μm-thick microtome sections were de-paraffinized. RPE melanin pigment was bleached by incubation for 2 min in fresh 1% sodium borohydrate solution before immunostaining. Sections were incubated in mouse anti-opsin N-terminus antibody clone B6-30 (a kind gift from Paul Hargrave, University of Florida, Gainesville, FL[32]) followed by AlexaFluor 488 conjugated secondary antibody (Life Technologies, Carlsbad, CA). DAPI was used to counterstain nuclei. Sections were mounted in Vectashield (Vector Laboratories, Burlingame, CA). Image z-stacks were acquired by recording x–y images 0.25 μm apart on a Leica TSP5 laser scanning confocal microscopy system. Opsin-positive phagosomes were counted in maximal projections of image stacks representing exactly 5-μm tissue depth and averaged per length of retina.

### Statistical analysis

The means ± standard errors were calculated for each comparison pair. *P*<0.05 was considered statistically significant for all experiments. Statistical analyses for ERG, qPCR, retinoid profile analysis, as well as pixel density quantification for immunostaining and Western analysis bands were performed in GraphPad Prism 6.0 (San Diego, CA, USA) using the Student's two-group, two-sided *t*-test. Comparison of ONL thickness (nuclei) was performed with one-way analysis of variance with post hoc pairwise comparisons using Bonferroni adjustment. Phagosome counts were evaluated using one-way ANOVA and Tukey’s post hoc tests to establish significant differences between time points.

## Results

### CD59a is expressed in mouse retina, RPE and choroid

**Fig 1:**
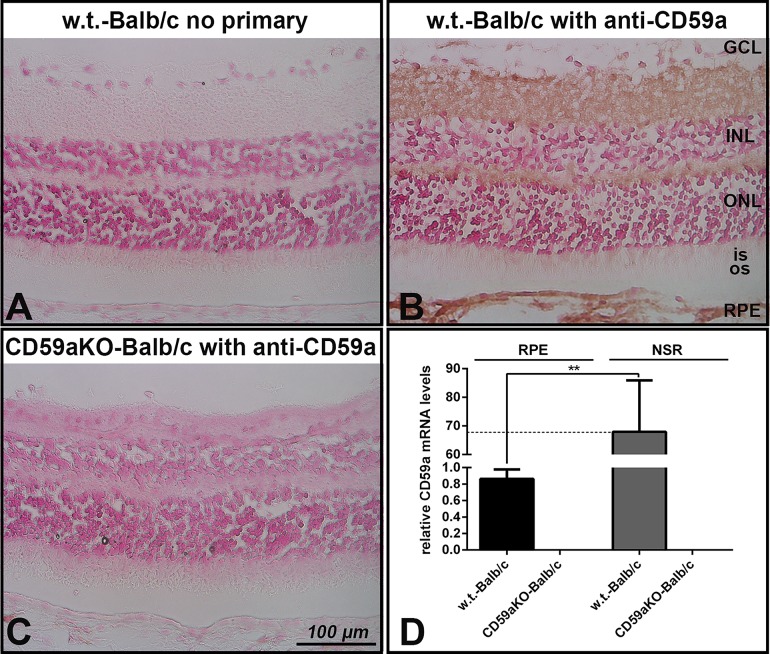
CD59a expression in mouse retina and mRNA levels of CD59a in NSR and isolated RPE. Photomicrograph showing CD59a immunolabeling in all layers of mouse retina, RPE and choroid (B), but not in CD59aKO eyes (C). Graph of qPCR results showing that mRNA levels of CD59a in NSR were more than 60-fold higher than that in isolated RPE cells in WT eyes (**P<0.001) (D). Data are expressed as means ± SD. N = 4. RPE, retinal pigment epithelium; OS, photoreceptor outer segment; IS, photoreceptor inner segment; ONL, outer nuclear layer; INL, inner nuclear layer; GCL, ganglion cell layer.

In the mouse, two CD59 genes have been identified [33–34]. CD59a is expressed broadly in various tissues, whereas CD59b is only expressed in the mouse testis [33–34]. Our immunostaining results showed that CD59a is present in all layers of NSR, as well as in the RPE and choroid (Fig 1B), but not in negative control CD59aKO retinas (Fig 1C). This localization is consistent with previously published results [35], now adding the KO control. The mRNA level of CD59a was more than 60-fold higher in NSR compared to isolated RPE cells of WT mice (*P =* 0.0003), but not detectable in CD59aKO samples (Fig 1D).

### CD59aKO mice are resistant to LD

**Fig 2:**
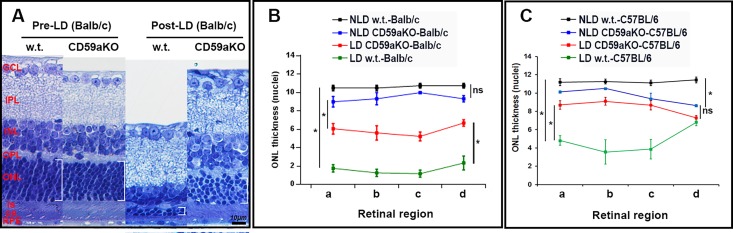
Photomicrographs of plastic sections of WT and CD59aKO retinas, and quantification of ONL nuclei. Histology of mouse retinas showing thinning of the ONL (white brackets) and IS/OS in post-LD WT retinas, while CD59aKO retinas showed less thinning of the ONL and IS/OS (A). Plot of the number of nuclei per column in the ONL. In WT retinas on both the Balb/c and C57BL/6 background, there was a significant decrease of ONL thickness across all sampled retinal regions (a-d) comparing non-light- damaged (NLD) (black line in B and C) and LD (green line in B and C). However, there was a smaller reduction of ONL thickness comparing NLD (blue line in B and C) and LD CD59aKO retinas (red line in B and C). Data are expressed as means ± SD. N = 4. *P<0.01, and significance markings refer to overall differences between groups across all four retinal regions (a-d).

Light damage experiments were performed in both Balb/c and C57BL/6J CD59aKO mouse strains. At day 7 following LD in Balb/c WT mice, we observed severe thinning of the ONL and loss of photoreceptor inner/outer segments (IS/OS). In comparison, Balb/c CD59aKO mice had a thicker ONL and better preserved photoreceptor IS/OS after LD (Fig 2A). Counting of ONL nuclei at four different retinal locations in the Balb/c strain revealed no significant difference in ONL thickness between non-light-damaged WT (NLD, black line in Fig 2B) and non-light-damaged CD59aKO mice (blue line) across all retinal regions a-d. In contrast, there were significantly fewer ONL nuclei in light-damaged WT retinas (LD, green line) compared to NLD WT (black line). Likewise, there were significantly fewer ONL nuclei in LD CD59aKO retinas (red line) relative to NLD CD59aKO (blue line). Comparing LD CD59aKO retinas (red line) to LD WT retinas (green line), there was partial preservation of ONL nuclei in the CD59aKO. In a parallel experiment with C57BL/6 mice, at day 10 following LD, we observed photoreceptor cell death in WT mice, which was mitigated in CD59aKO retinas (Fig 2C). However, in contrast to the non-pigmented Balb/c strain, there was a small but significant reduction in ONL thickness in C57BL/6 NLD CD59aKO mice (blue line in Fig 2C) compared to NLD WT (black line).

### Retinal ERG responses in CD59aKO mice are reduced before LD but preserved after LD

**Fig 3:**
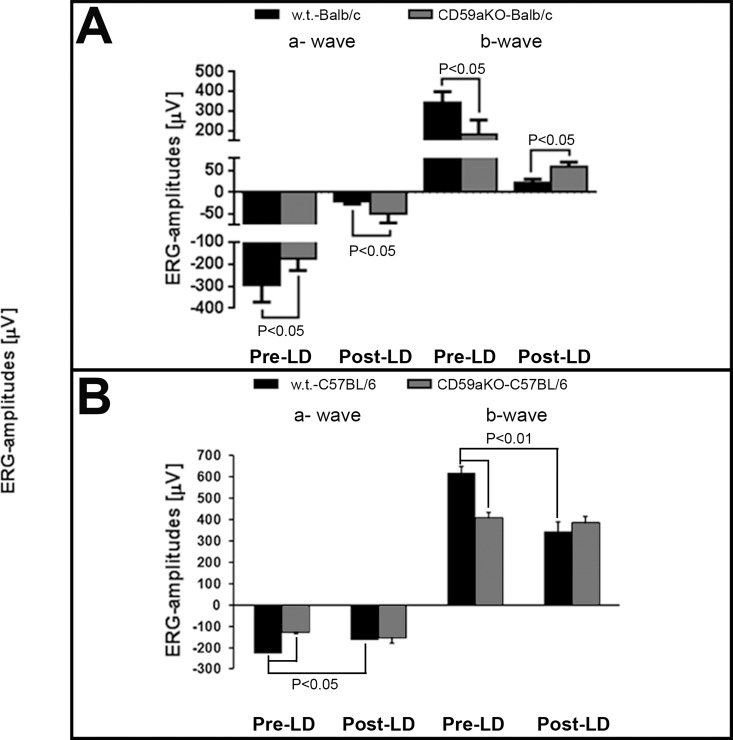
ERG responses before and after LD. Before LD, rod-a (*P*<0.05) and rod-b (*P*<0.05) waves were decreased in CD59aKO Balb/c mice compared to WT (A). At day 7 after LD, however, rod-a (*P* = 0.04) and rod-b (*P* = 0.01) waves were all larger in CD59aKO compared to WT (A). Data are expressed as means ± SD. N = 4. Similarly, rod-a (*P*<0.05) and rod-b (*P*<0.01) wave amplitudes were smaller in CD59aKO C57BL/6J mice compared to C57BL/6J mice at baseline, and while C57BL/6J mice have diminished ERG amplitudes 10 days after LD, amplitudes in CD59aKO mice remain unchanged (B). Data are expressed as means ± SD. N = 6–7.

Full-field ERG was employed to evaluate retinal function in the two CD59aKO strains. In both genetic backgrounds, pre-light exposure CD59aKO mice showed reduced rod a- and rod b-wave amplitudes compared to their respective pre-LD WT (Fig 3A and 3B). At day 7 after LD, however, Balb/c CD59aKO mice had significantly higher amplitude rod a and b-waves relative to WT mice (Fig 3A). Consistent with results from the Balb/c strain, there were no differences in ERG responses of C57BL/6 CD59aKO 10 days after LD compared to the significant reduction in C57BL/6 WT mice (Fig 3B). This observed ~50% reduction in ERG amplitudes in pre-LD CD59aKO C57BL/6 mice relative to WT is unlikely to have been caused exclusively by the thinning of the ONL in NLD CD59aKO mice (Fig 2C).

### Rhodopsin levels and 11-cis retinal regeneration in CD59aKO mice

**Fig 4:**
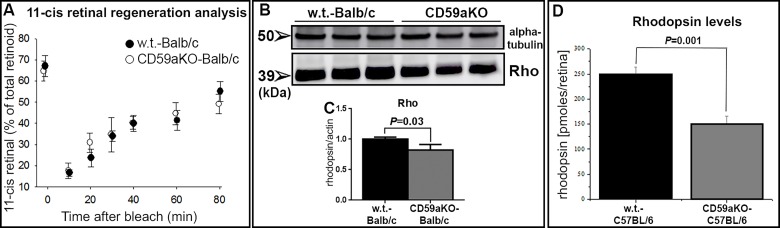
11-cis retinal regeneration and rhodopsin levels in CD59aKO retinas. 11-*cis* retinal regeneration analysis (A). Before bleaching, there was no difference in the percentage of 11-cis retinal of total retinoid. Also, no differences were observed in regenerated 11-*cis* retinal at 10 m intervals after bleaching as indicated. Data are expressed as means ± SD. N = 8. Western blotting analysis of rhodopsin protein in WT and CD59aKO-Balb/c mice (B and C). There was significantly lower rhodopsin protein in CD59aKO mice compare to WT-Balb/c mice (*P* = 0.03). N = 3. Microspectrophotometric rhodopsin measurement in NSR of C57BL/6 WT and CD59aKO (D). There was significantly lower rhodopsin concentration per retina in CD59aKO mice compared to WT C57BL/6 mice (*P* = 0.001). N = 5.

Anatomic and functional protection against light damage can be due to significantly slower 11-*cis* retinal regeneration or a reduction in visual pigment levels (i.e., less substrate for light to interact with and cause phototoxicity). To examine these possibilities, 11-*cis-*retinal regeneration as a percentage of total retinoids was measured at several time points after bleach in Balb/c CD59aKO mice (Fig 4A, S1 and S2 Tables). No difference was observed in regeneration between CD59aKO and WT, implying that the visual cycle was not affected by loss of CD59a. Next, rhodopsin protein levels in Balb/c WT and CD59aKO mice were measured via Western-blotting analysis (Fig 4B), and rhodopsin levels in Balb/c CD59aKO retinas were significantly lower relative to WT (Fig 4C, *P* = 0.03). Rhodopsin concentration per retina was also measured by microspectrophotometry in C57BL/6 mice (Fig 4D). Consistent with results of Western analysis from Balb/c mice, C57BL/6 WT mice contained 250 ± 13.1 pmol of rhodopsin per retina while CD59aKO mice contained only 150 ± 16.5 pmol (*P* = 0.001).

### RPE phagocytosis of spent photoreceptor outer segment fragments is normal in CD59aKO mice

**Fig 5:**
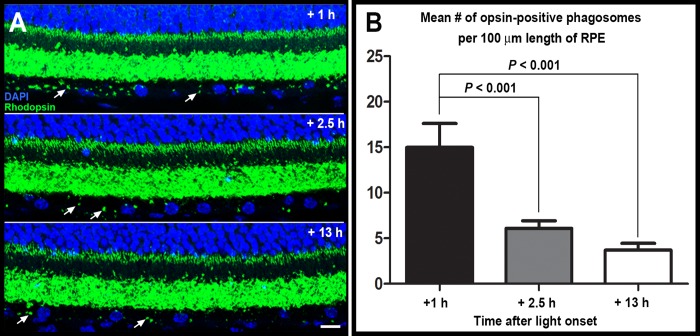
Quantification of RPE phagocytosis of shed outer segment fragments via immunostaining with anti-rhodopsin antibody. CD59aKO RPE showed maximal phagosome content 1 h after light onset as characteristic for normal outer segment renewal. Representative retinal cross sections (A) showing opsin immunolabeling (green) and cell nuclei (blue) of CD59aKO outer retina of mice sacrificed at 1 h, 2.5 h, and 13 h after light onset as indicated. In each section, two phagosomes in the RPE are indicated by arrows. Scale bar = 10 μm. Quantification of phagosome counts (B). Eyes from 5 different mice for each time point, and 6 sections of each eye were imaged for each mouse. Data are expressed as mean ± SD. N = 5.

Long-term photoreceptor cell function relies on continuous photoreceptor outer segment renewal, which is dependent on a balance between outer segment growth and shedding of distal outer segment tips. Shed photoreceptor outer segments are phagocytosed by RPE cells in a diurnal fashion, peaking shortly after light onset. Outer segment renewal can be assessed by quantifying the phagosome load of the RPE, which was assessed in this study by immunostaining tissue sections with an antibody against rhodopsin (Fig 5A). Here, we quantified the frequency of rhodopsin-positive phagosome inclusions within the RPE as a function of time of day. C57BL/6 CD59aKO mice showed a clear peak of RPE phagosome load 1 h after light ON, with levels returning to baseline by 2.5 h after light ON, and maintained at 1 h after light OFF (i.e., 13 h after light ON) (Fig 5B). These results suggest that outer segment renewal takes place and follows a diurnal rhythm in CD59aKO mice as is characteristic for WT mice with a normal photoreceptor outer segment renewal program [36].

### GFAP is up-regulated in CD59aKO retinal Müller cells

**Fig 6:**
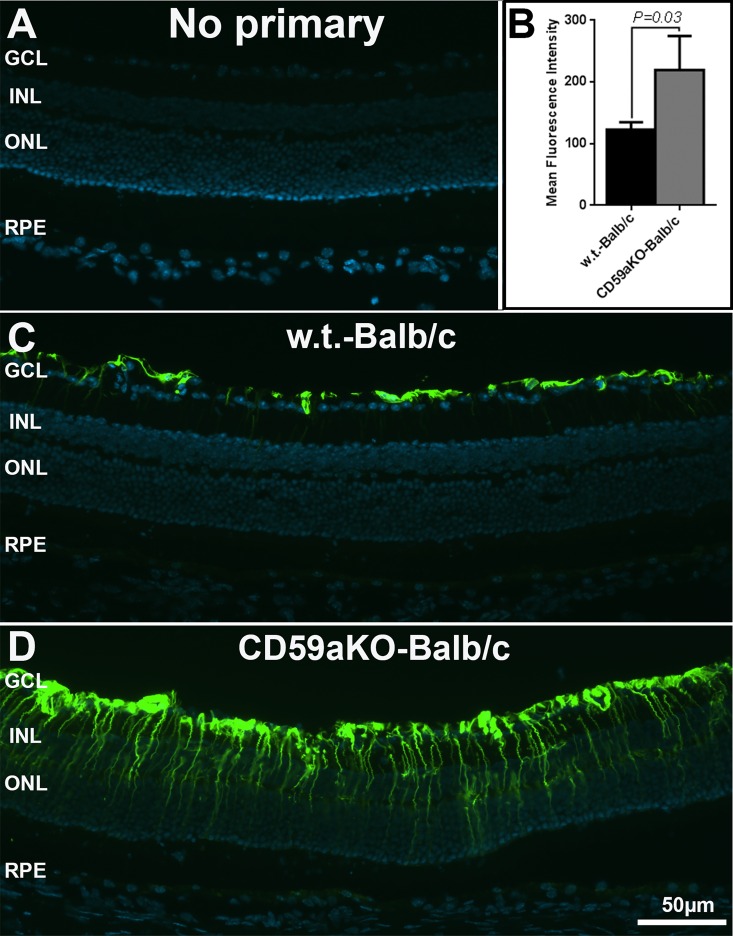
Immunofluorescence microscopy of GFAP in NLD CD59aKO and WT retinas. Compared to the labeling limited to the innermost retina in WT mice (C), the GFAP signal was up-regulated in Müller cells of CD59aKO retinas (D). Quantification of fluorescence intensity showed that GFAP was significantly increased in CD59aKO retinas compared to controls (B) (*P* = 0.03). Data are expressed as means ± SD. N = 4.

Protection against light damage could also be due to preconditioning generated by the gene defect, a phenomenon we have observed in the past in brain-derived neurotrophic factor (BDNF) knockout mice [37]. In order to assess the potential for Müller cell activation by CD59aKO, NLD retinal sections were immunostained with an anti-GFAP antibody. In WT retinas, specific GFAP staining was confined to the retinal nerve fiber layer astrocytes (Fig 6, compare C to A). However, GFAP was increased in Müller cells of CD59aKO retinas (Fig 6D). Measurement of pixel intensity confirmed higher GFAP levels in NLD CD59aKO retinas compared to NLD WT controls (Fig 6B, *P* = 0.03).

### Neurotrophic factors and ER-stress are up-regulated in CD59aKO retinas

**Fig 7:**
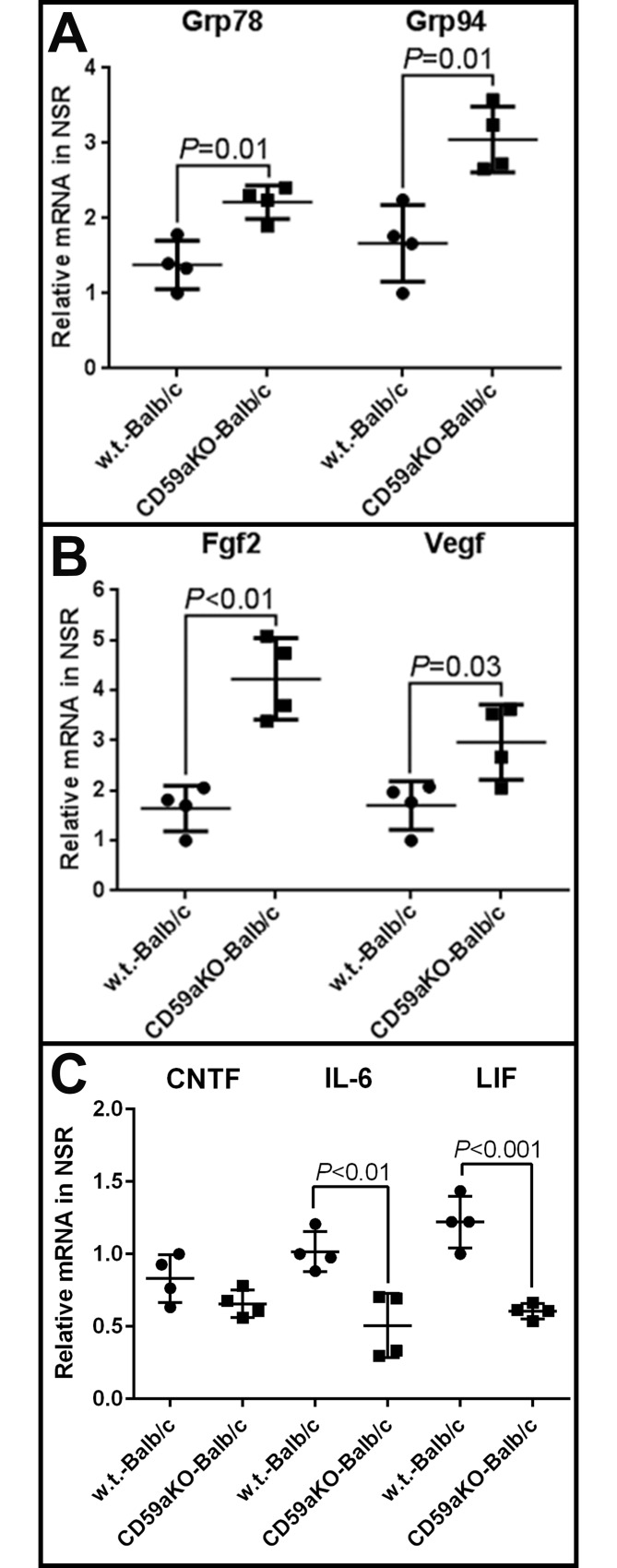
The mRNA levels of ER-stress genes and neurotrophic factor genes in NLD CD59aKO retinas versus WT retinas. Two ER-stress genes, Grp78 (*P* = 0.01) and Grp94 (*P* = 0.01), were significantly up-regulated in CD59aKO retinas (A). Neurotrophic factor genes, Fgf2 (*P*<0.01) and Vegf (*P* = 0.03), also were significantly up-regulated in CD59aKO retinas (B). mRNA levels were also measured for three members of the neuropoietic cytokine family known to have retinal neuroprotective effects: ciliary neurotrophic factor (CNTF), interleukin-6 (IL-6), and leukemia inhibitory factor (LIF). mRNA levels for all three factors appeared lower in CD59aKO relative to WT retinas, with significant differences in IL-6 (*P*<0.01) and LIF (*P*<0.001) expression (C). Data are expressed as means ± SD. N = 4.

**Fig 8:**
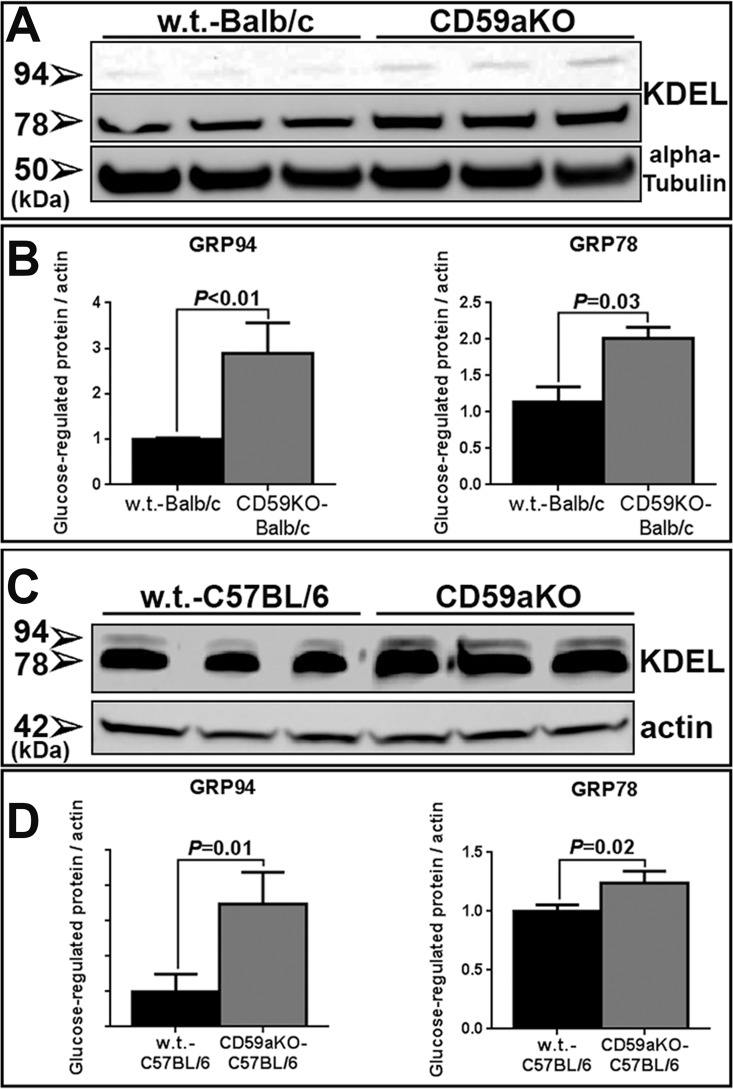
Western blotting analysis of heat shock proteins in NLD CD59aKO versus WT retinas. The anti-KDEL antibody recognizes both GRP78 and GRP94, and they were both increased in retinas of CD59aKO-Balb/c mice (A). Comparison of the levels of GRP78 (*P* = 0.02) and GRP94 (*P* = 0.01) showed higher levels in CD59aKO (B). Similarly, both GRP78 (*P* = 0.03) and GRP94 (*P*<0.01) were increased in retinas of CD59aKO-C57BL/6 mice (C and D). Data are expressed as means ± SD. N = 3.

We further assessed retinal stress by determining levels of ER-stress markers via qPCR. Two ER stress markers, Grp78 and Grp94, were both up-regulated in NSR from Balb/c NLD CD59aKO mice compared to NLD WT (Fig 7A). Neurotrophic factors Fgf2 and Vegf were also up-regulated in Balb/c NLD CD59aKO retinas (Fig 7B). Consistent with the qPCR result, GRP78 and GRP94 protein levels were also significantly higher in NLD CD59aKO retinas compared to levels in WT retinas in both Balb/c (Fig 8A and 8B) and C57BL/6 mice (Fig 8C and 8D).

To elucidate the mechanism of reduction in ERG amplitudes and rhodopsin expression in NLD CD59aKO retinas, we considered changes in expression of various factors. Ciliary neurotrophic factor (CNTF) has been shown to decrease ERG amplitudes and rhodopsin expression when up-regulated [38]. In addition, interleukin-6 (IL-6) and leukemia inhibitory factor (LIF) are members of the same neuropoietic cytokine family as CNTF with similar retinal neuroprotective effects observed in other contexts [39–40]. In our study, the mRNA levels for all three factors appeared lower in CD59aKO relative to WT retinas, with significant differences in IL-6 (*P*<0.01) and LIF (*P*<0.001) expression (Fig 7C).

## Discussion

Herein, we found that CD59a is expressed in all layers of the NSR, and also in the RPE and choroid. In the absence of CD59a, photoreceptor sensitivity to light is reduced and the retina undergoes a stress response indicated by increased levels of ER-stress gene (Grp78 and Grp 94) mRNAs, Müller cell GFAP expression, and neurotrophic factor (Ffg2 and Vegf) expression. These responses to CD59aKO are associated with decreased photoreceptor susceptibility to LD. This diminished LD in CD59aKOs was discovered independently in two separate labs on two genetic backgrounds (BR using C57BL/6 and JLD using Balb/c). Regarding differences between the two strains, C57BL/6 mice are more resistant to LD because the pigment in the RPE cells exerts a protective effect by absorbing light and because the RPE65met450 isoform in this strain regenerates 11-*cis*-retinal more slowly. Our observation that, unlike in the Balb/c strain, there was no significant decrease in ONL thickness after LD in C57BL/6 CD59aKO mice (Fig 2C) is consistent with this phenomenon.

Previous studies have shown that CD59a is expressed in both human and mouse retinas and RPE cells [35,41,42]. Our results reproduced these findings and, in addition, showed that CD59a mRNA levels in the NSR were more than 60-fold higher than in RPE cells. These results suggest CD59a may play an important MAC-inhibitory role in the NSR.

In this study, we found that CD59aKO mice at baseline (before LD) had lower ERG amplitudes than their age-matched and genetic background-matched controls. This reduction in ERG amplitudes was, however, not due to a significant loss of photoreceptors or abnormality in photoreceptor outer segment renewal, but rather due to reduced sensitivity of the photoreceptors to light. CD59aKO rod photoreceptors exhibited lower levels of rhodopsin, but important RPE functions in support of photoreceptors such as 11-*cis* retinal regeneration and diurnal RPE processing of phagocytosed outer segment tips were normal, suggesting that the loss of CD59a may have caused changes in the NSR rather than the RPE.

In support of the hypothesis that some optimal level of complement activation is required for full retinal functionality, eliminating both activators and inhibitors in the complement cascade have been shown to decrease retinal function. Previously, we have shown that in mice lacking complement factor B (a required activator of the alternative pathway of complement), both rod and cone ERG amplitudes were reduced by 20–30% across all light intensities by 9 months of age [43]. In these mice, there was no loss of cells in the ONL or INL, but rather a reduction in opsin and RPE65 gene expression. Similar reductions in ERG amplitudes have been reported for mice lacking the anaphylatoxin C3a receptor (a G-protein-coupled receptor required for chemotaxis) and complement component C3 (a central component required for all three complement activation pathways) [44]. Finally, elimination of complement factor H (an inhibitor in the alternative pathway) has resulted in significantly reduced visual acuity and rod ERG amplitudes in aged mice [45]. It will be of great interest to determine the beneficial effects of optimal complement activation in ocular tissues in the context of different genetic backgrounds on tissue homeostasis, retinal structure, function, and pathology [46].

As discussed in the recent review by Wenzel and colleagues, light-damage induced by short exposures to bright white light (as was used in our experiments) requires the visual pigment rhodopsin as an essential trigger of the cell death mechanism [47]. Hence, the reduced amounts of bleachable rhodopsin in the presence of normal rates of pigment regeneration observed in the CD59aKO mice could be responsible for their reduced sensitivity to light damage.

The search for neuroprotective agents for photoreceptors has uncovered many pathways and mechanisms that play a role in this process. One of those mechanisms is pre-conditioning. Liu and colleagues [48] have demonstrated that exposure of animals to 12 hours of bright light 48 hours prior to bright light damage significantly protected the rat retina from photoreceptor degeneration. This preconditioning was correlated with an increase in neuroprotective factors Fgf2 and CNTF. In agreement, mice with reduced levels of the neurotrophic factor BDNF (BDNF^+/-^) are protected against light damage [37]. These mice have increased levels of glial-derived neurotrophic factor (GDNF) mRNA prior to LD, and have shown preservation of neurotrophin levels after LD. We speculated in that manuscript that “chronic reduction of BDNF in the retina provides a level of preconditioning against stress”. Our findings in the present study of increased mRNA levels and proteins indicative of ER stress, Müller cell GFAP, and neurotrophic factors such as FGF2 in CD59aKO retinas further support this notion of constitutive retinal preconditioning. To further explore the mechanism of preconditioning, we considered the following members of the neuropoietic cytokine family: CNTF, IL-6, and LIF. None of the three factors was up-regulated in CD59aKO retinas, suggesting that they are not responsible for the observed decrease in ERG amplitudes and rhodopsin expression in the CD59aKO mice.

CD59a is a MAC-inhibitory protein, also called membrane inhibitor of reactive lysis. It prevents C9 from binding to the preformed C5b-8 complex on the cell membrane, and hence prevents formation of the final complement membrane attack complex. Although we cannot measure MAC directly in our study due to unavailability of effective MAC detection assays suitable for mice, we hypothesize that the absence of CD59a leads to increased MAC formation. Prolonged or high levels of MAC formation may result in cell lysis, whereas short or low levels of MAC formation may result in sub-lytic effects. However, the lack of retinal degeneration in the CD59aKO mouse may serve as yet another example of redundancy in the complement system. In the absence of CD59a, other inhibitors of the terminal pathway appear to limit MAC activation. Those might include protein S, a vitamin K-dependent plasma glycoprotein [49], and clusterin [50], a protein also known to be associated with the clearance of cellular debris and apoptosis [51]. Interestingly, clusterin expression is increased in light-induced retinal degeneration [52].

How might sub-lytic activation of complement in the absence of CD59a lead to changes in photoreceptor cell function and subsequent protection against light damage in the NSR? Previous studies in vascular endothelial cells revealed that MAC-induced reversible changes in cell membrane permeability lead to altered concentrations of cytosolic ions. These changes, in turn, lead to cellular proliferation triggered by release of growth factors [53–54]. Similarly, cell-membrane pore formation by sub-lytic MAC on photoreceptors may lead to sodium and calcium ion influx, partly depolarizing (light-adapting) the photoreceptor, which could limit light-induced hyperpolarization. This could be a reason for reduced ERG response in NLD CD59aKO retinas.

Studies on the effects of sub-lytic MAC on the plasma membrane of the RPE suggest that MAC can also affect the behavior of this cell type. Sub-lytic MAC assembly on RPE cells generates a pro-inflammatory microenvironment, including increased RPE production of IL-6 [14,55,56]. In photoreceptors, rhodopsin levels are diminished by the IL6 related cytokine leukemia inhibitory factor (LIF) [57]. Thus, MAC-induced IL-6 upregulation may contribute to the observed rhodopsin down-regulation in CD59aKO mice.

In summary, our data highlight the importance of CD59a for retinal function. They also indicate that stress caused by CD59aKO affords partial protection against an acute insult in the form of damaging light. This protection may result from diminished rhodopsin levels, upregulation of stress response genes, or both.

## Supporting Information

S1 Table The absolute retinoid content (picomoles/eye) measured in the dark was unchanged in Balb/c CD59aKO mice relative to WT. Data are expressed as means ± SD. N = 8.
(TIF)

S2 Table The relative percent of retinoid content (picomoles/eye) measured in the dark was unchanged in Balb/c CD59aKO mice relative to WT. Data are expressed as means ± SD. N = 8.
(TIF)

## Abbreviations

AMD: age-related macular degeneration
KO: knockout
ER: endoplasmic reticulum
Rho: rhodopsin
LD: light damage
GPI: glycosylphosphatidylinositol
MAC: membrane attack complex
WT: wild-type
PFA: paraformaldehyde
GFAP: glial fibrillary acid protein
NSR: neurosensory retina
qPCR: quantitative PCR
ERG: Electroretinography
NLD: no light damage
RPE: retinal pigment epithelium
BDNF: brain-derived neurotrophic factor
Fgf2: fibroblast growth factor 2
Vegf: vascular endothelial growth factor
Grp: glucose regulated protein
CNTF: ciliary neurotrophic factor
IL-6: interleukin-6
LIF: leukemia inhibitory factor
GDNF: glial-derived neurotrophic factor
OS: photoreceptor outer segment
IS: photoreceptor inner segment
ONL: outer nuclear layer
INL: inner nuclear layer
GCL: ganglion cell layer
