# Supplementary figures and images for: Retinal Pre-Conditioning by CD59a Knockout Protects against Light-Induced Photoreceptor Degeneration
Source: PLoS One. 2016 Nov 28;11(11):e0166348. doi: 10.1371/journal.pone.0166348 (PMC5125596; doi:10.1371/journal.pone.0166348)

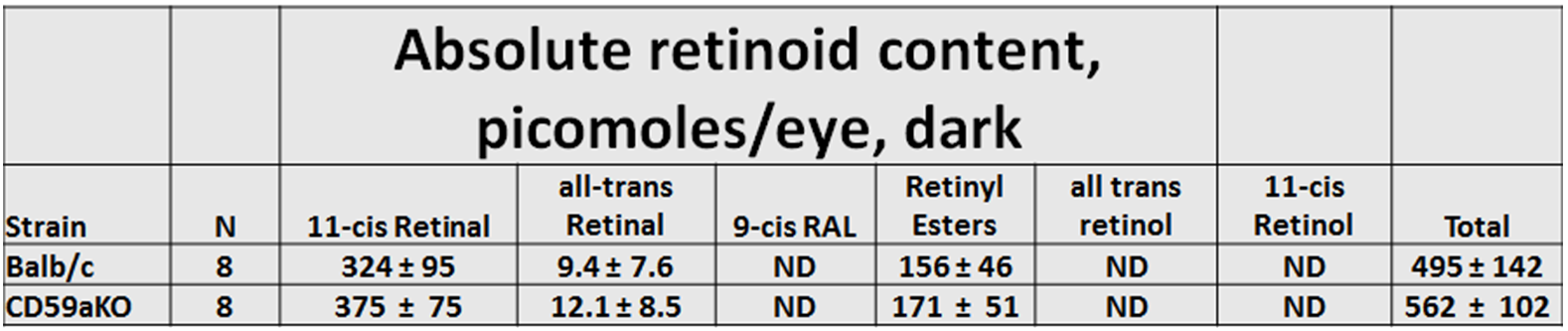

Supplement: S1 Table — Data are expressed as means ± SD. N = 8. (TIF) [file pone.0166348.s001.tif]

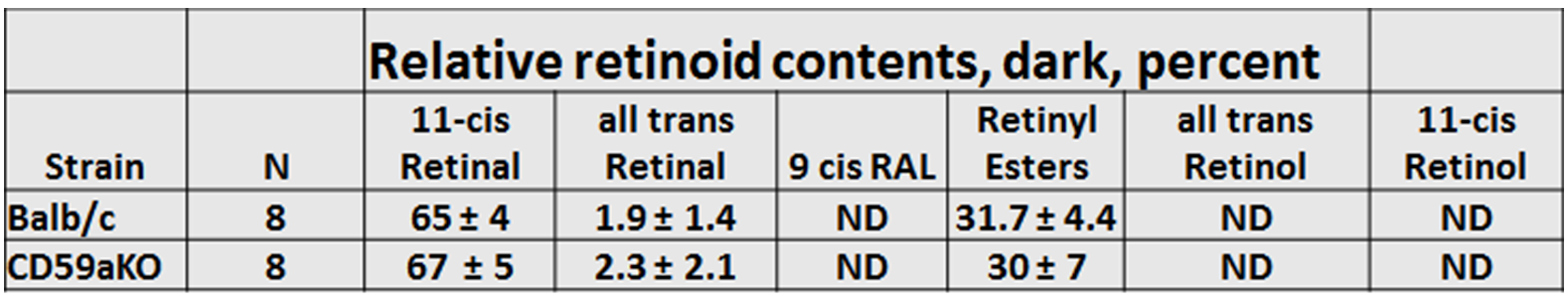

Supplement: S2 Table — Data are expressed as means ± SD. N = 8. (TIF) [file pone.0166348.s002.tif]
